# 

**Published:** 1911-06-24

**Authors:** 


### MEDICAL APPOINTMENTS.

The Election Committee of the Liverpool Royal Infirmary has appointed Mr. T. C. Litler Jones, F.R.C.S.Eng., as honorary surgeon to the institution, to fill the vacancy created by the retirement of Mr. F. T. Paul, whose term of office has expired. Mr. Litler Jones became an honorary assistant surgeon in 1903, and has been senior assistant surgeon since 1908.

